# The Logic of Appearance: Dennett, Phenomenology and Psychoanalysis

**DOI:** 10.3389/fpsyg.2017.01437

**Published:** 2017-08-22

**Authors:** Jasper Feyaerts, Stijn Vanheule

**Affiliations:** Department of Psychoanalysis and Clinical Consulting, Ghent University Ghent, Belgium

**Keywords:** Dennett, phenomenology, psychoanalysis, subjectivity, experience, belief

## Abstract

In the present essay, we aim to develop and contrast three different positions toward Sellars’ distinction between the manifest and scientific images of man: Dennett’s philosophical reconstruction of neurocognitive science, contemporary phenomenology and psychoanalysis. We will suggest that these respective traditions and the substantial differences between them can be understood in terms of a ‘logic of appearance.’ Related to this are differing ideas about the rights and limits of the first-person perspective, the relation between conscious experience and belief, and the issue of naturalization. In the final part, we will try to specify, on the basis of a detailed reading of the disagreement between Dennett and phenomenology, in what way psychoanalytic theory could respond to these different issues.

## Introduction

In his *Philosophy and the Scientific Image of Man*, Wilfrid Sellars offered a condensed diagnosis that seems to retain a certain validity with respect to our current philosophical, scientific and social predicament. Sellars (1991) famously talked about a discord fueled by the conflict between two apparently opposing “images” of man-in-the-world (1991, p.5): on the one hand, a *manifest image* which represents the tentative and provisional collection of ideas and assumptions we spontaneously adopt to characterize ourselves and the world we live in. Chief among these are ideas closely related to our immediate self-understanding as subjects endowed with self-consciousness, a certain extent of freedom of choice, gifted with reason but also desires, and so on. On the other hand, a relatively recent, but continually expanding *scientific image* of man as a “complex physical system” (1991, p. 25) – that is, an image conspicuously *unlike* its manifest double, but one which can be progressively distilled from various burgeoning scientific disciplines, such as neurology, physics, evolutionary biology and cognitive science.

In a more recent past, Sellars’ distinction has found a renowned application in the work of the well-known philosopher of cognitive neuroscience, Daniel Dennett. For the past few decades, Dennett has been vehemently advocating against “the fantasy of first-person science” (Dennett, 2001), arguing, amongst other things, that there is no such thing as a ‘Cartesian theater’ where private things happen that are spontaneously revealed before a ‘mental I/eye’, one that, moreover, would form an unsurpassable source for self-knowledge from a first-person perspective. Why indeed, Dennett asks, should we give up our scientific abstinence when, compared to some other notorious fictive entities such as the phlogiston or élan vital, it comes to seemingly less exotic matters such as the ‘I’, ‘experience’ and ‘consciousness’? According to Dennett’s well-known proposal, we should treat these manifest testimonies in the same way we treat testimonies in court, that is, as provisional *fictions* (Dennett, 1991, p. 79), where after we can safely let cognitive neuroscience do its job in deciding if, and to what extent, there might be “truth in fiction.”

Given the central importance of the first-person perspective – in one way or another, and to be specified throughout our article – in both contemporary phenomenology and psychoanalytic theory, the questions we want to pose in this article are therefore the following ones: where do these disciplines, respectively, stand with regard to such proposals? And in what way would these two disciplines respond to Dennett’s proposal to consider subjective beliefs about ourselves as provisional fictions and, to be sure, in all likelihood, future illusions?

In the first part, we will give a detailed reading of what is understood by such terms as ‘first-person experience’ and ‘subjective appearance’ in both phenomenology and Dennett’s account and, on the basis of that reading, press some arguments that can be advanced against Dennett’s fictionalization of subjectivity in favor of phenomenology. In the second part, we will offer a similar exercise in the case of psychoanalysis. We will argue that Sellars’ distinction, exemplified by the discussion between Dennett and phenomenology, touches upon a classic ambiguity in psychoanalytic theory. Finally, a possible way out of this ambiguity will be sought through a close examination of the psychoanalytic concept of ‘belief’.

## Dennett and Phenomenology: The Seeming of Seeming

Let us take as a point of entry in the already quite extensive debate between phenomenology and Dennett^1^ one of the latter’s most cherished examples which he uses in his *Consciousness Explained* in order to fortify his Multiple Drafts Model of consciousness, a model which, Dennett warns us, “is initially deeply counterintuitive” (1991, p. 45). Dennett refers to a certain perceptual effect, better known in the literature as the *phi phenomenon* or *phi movement*, first so called by the Gestalt psychologist Max Wertheimer, which was the object of repeated study in a series of experiments conducted by Kolers and von Grünau (1976). In the simplest case of the experiment, subjects were shown a single flash of a red spot, followed quickly by a displaced green spot, followed by another flash of the original red (each flash lasting about 150 ms, with a gap of about 50 ms in between). Perhaps not that surprising given our everyday smooth television-experience, the subjects reported seeing not two discrete spots, but one spot rapidly moving over and back again. However, one might ask, what about the change of color from red to green? Interestingly, the subjects reported the spot changing color about halfway through its trajectory from the initial flash to the second. As Dennett (1991) recounts the result: “the first spot seemed to begin moving and then change color abruptly in the middle of its illusory passage toward the second location” (p. 114). Given this surprising result, one of the collaborators to the experiments, the philosopher Nelson Goodman, asked the following question: “how are we able to fill in the spot at the intervening place-times along a path running from the first to the second flash *before that second flash occurs*” (Goodman, 1978, p. 73)? A pressing question indeed in so far as we want to avoid positing strange ontological scenarios wherein effects precede their causes and time starts running backward.

So how then are we to proceed in answering Goodman’s question? We will first begin by teasing out some basic principles in the way phenomenology would handle this issue, in order to articulate more clearly the dividing line which separates Dennett’s account from the former.

### The Principle of Appearance Qua Appearance

From a phenomenological perspective, when confronted with such an ambiguous situation, it would be wise to make use of Husserl’s notorious maxim of phenomenological inquiry and “to go back to the things themselves” (Husserl, 2001, p. 168). Indeed, one of the guiding motifs and enabling assumptions of phenomenology is that *in principle*, and through availing ourselves of phenomenology’s methodological procedures of ‘epoché’ and ‘reduction’, we would be able to put these explanatory questions aside for a moment and to focus instead on the experienced object (in this case the moving spot) precisely in the way it is experienced from the subjective or first person point of view. Rather than the hasty conjuring up of various theoretical explanations (e.g., Goodman’s “how are we able to fill in the gaps?”), phenomenology’s traditional first move is to get a clear descriptive picture of what is to be explained in the first place – the *quod erat explicatum* for a science of consciousness -, precisely by letting, to use Heidegger’s inimitable phrasing, “that which shows itself be seen from itself in the very way in which it shows itself from itself” (Heidegger, 1962, p. 34). Despite the different ways in which contemporary phenomenology aims to fulfill its role in the debate around the nature of consciousness, there is indeed at least one unifying principle amongst the various phenomenological commentators. Let us call this the phenomenological principle of *appearance qua appearance*. In order to get a clear view of the sort of claims at stake in such a principle, let us begin by considering a few remarks by Husserl. In the *Crisis of European Sciences and Transcendental Phenomenology*, Husserl (1970) writes:

The first thing we must do, and first of all in immediate reflective self-experience, is to take conscious life, completely without prejudice, just as what it quite immediately gives itself, as itself, to be (p. 233).

Here we have the first point: if anything, phenomenology, as a transcendental investigation of the first-person perspective, takes its point of departure “in immediate self-experience”, that is, it tries to offer a descriptive analysis of the way the world in general (Wertheimian spotlights included), but also other subjects and we ourselves, appear to us in ordinary experience. Crucially, in offering such a description of our everyday intentional involvement with the world, this appearance should be accepted “just as what it quite immediately gives itself, as itself, to be” (Husserl, 1970, p. 233). In short: on a phenomenological account, appearance should be taken as appearance *as such*, rather than as something else. Let us take one of Husserl’s examples to clarify and develop the ramifications of such an idea a little bit further.

Suppose we walk through a garden and become struck by a blossoming tree. How do we go about analyzing our immediate experience of the appearing of this blossoming tree? It is here that Husserl’s methodological tool of ‘epoché’ comes into play; a notion, however, particularly fraught with all kinds of misunderstandings we need to clear up before furthering our analysis (see Overgaard, 2015). According to a common misunderstanding, for example, the method of epoché or ‘bracketing’ would lead us to a consideration of the immanent contents of perception as they show up ‘in’ consciousness, severed and disconnected from any possible relation to the transcendent, real world which, conversely, is considered to be ‘outside’ consciousness (e.g., Burge, 2010). Accordingly, phenomenological bracketing would be first of all the bracketing of the realist presumption inherent in the natural attitude of our everyday experience. That is, the elimination of our pre-philosophical belief in the real world in favor of the appearance of Husserl’s blossoming tree as some kind of second blossoming tree, this time considered as an immaterial entity (i.e., image, datum, perceptum, …) somehow hovering before or within consciousness as in a Dennettian Cartesian theater. Yet closer inspection of some of Husserl’s own remarks on what the epoché is supposed to accomplish makes it clear that such an introspective reading is simply mistaken. Remarking on how that blossoming tree appears to consciousness after the epoché has been performed, Husserl (1983) writes

[…] everything remains as of old. Even the phenomenologically reduced perceptual mental process is a perceiving *of* “this blossoming apple tree, in this garden,” etc., and, likewise, the reduced liking is a liking of this same thing. The tree has not lost the least nuance of all these moments, qualities, characteristics *with which it was appearing in this perception*, <with which > it < was appearing as > “*lovely*,” “*attractive*,” and so forth “in” *this liking* (p. 216).

Hence, the practice of phenomenological bracketing as originally intended by Husserl does not consist in a reflexive retreat from reality the better to focus on contents or ‘qualia’ that are found within the self-enclosed terrain of pure consciousness. Nevertheless, although everything after the epoché “remains as of old” (Husserl, 1983, p. 216), there is of course a sense in which the epoché, in Husserl’s understanding, does reveal something that might have gone unnoticed before we put it to use. Again, Husserl (1970) remarks:

[…] through the epoché a new way of experiencing, of thinking, of theorizing, is opened to the philosopher; here, situated above his own natural being and above the natural world, he loses nothing of their being and objective truths and likewise nothing at all of the spiritual acquisitions of his world-life (…); he simply forbids himself – as a philosopher […] – to continue the whole natural performance of his world-life (p. 152).

Two points are important in this rendition of the epoché.

The first thing to emphasize is that this new way of experiencing is not a matter of acquiring *additional* empirical information through further forms of experience, for example, by turning my introspective gaze from the world to my consciousness. This means that, with regard to the earlier example of Husserl’s blossoming tree, the epoché is intended to enable a faithful attending and description of the original experience of the blossoming tree precisely as it appeared in that very experience, that is, without changing anything about all the moments and elements that were already implicit in that experience. So if in our straightforward experience of the blossoming tree, it appeared as ‘actually existing’ or ‘real’, mind independent in whatever sense, but also ‘lovely’, ‘attractive’, ‘in this garden’ and so forth, then these presuppositions, and basically all positions with regard to that tree, should be retained in our description.

Yet, secondly, in this reflexive elucidation of conscious experience through the epoché, not only does the focus shift from world to world-as-experienced, but so does the criterion for *truth* and *error* with regard to phenomenological statements that try to express descriptively what was going on according to experience. That is, by abstaining from taking any positions toward the conscious experience of, for example, spotlights changing color at a certain moment, we seem to be moving to what is at least a different sort of epistemological terrain when compared to the epistemic situation of empirical claims about the world (see Rosenberg, 2002). Yet, we should be careful as to the precise nature of the epistemic shift that, according to phenomenology, is at stake here.

Does such a claim, for example, imply some sort of “papal infallibility” (Dennett, 2007, p. 6) or incorrigibility with regard to phenomenological beliefs relative to the first-person perspective? Here is how Koch (1997) formulates such an idea:

We shall take the *cogito* only as a means of suspending objectivity claims and of thereby inducing infallibility in what remains of the objectivity claim after suspension. This last point is important. For every objective truth claim, in which I am invariably fallible, there is a corresponding trivial truth claim, in which I am infallible, a truth claim which is fulfilled by the sheer fact that I seriously and honestly claim so. For every objective, thick truth claim, that p, there is a corresponding trivial, thin truth claim, that I think that p (or that it seems to me that p) (p. 73).

Hence, on Koch’s reading (but also Dennett’s, cf. infra), the epoché entails a movement from objectivity claims in which I am invariably fallible, e.g., the explicit judgment ‘the spot changes color about halfway its trajectory’, to a *suspension* of the same objectivity claim for which in that case I become infallible, ‘it seems to me that the spot changes color about halfway its trajectory’. Moreover, the reason why such a transition from judgment to suspension implies infallibility is because in this case, according to Koch, the cogito “falls within its own scope” (1999, p. 72). What this means is that the cogito, represented by the expression ‘I think that p’ or ‘it seems to me that p’, cannot be iterated without becoming trivial since an additional deployment of the epoché would add no further relevant information beyond what can already be found after the first suspension: ‘I think that p’ is logically equivalent to ‘I think that I think that p’ or ‘it seems to me that it seems to me that p’.

However, it is highly questionable whether Husserl and other major figures in the phenomenological tradition would apprehend the outcome of the epoché and the suggested first-person incorrigibility in such an intellectualistic and Cartesian way. Here, the question is: should we understand the phenomenological transition from world to world-as-experienced as a suspension from objectivity claims to subjectivity claims in terms of a regression to the ‘I think’ of the Cartesian cogito? Husserl (1970), for one, does not seem to endorse this reading:

It is naturally a ludicrous, though unfortunately common misunderstanding, to seek to attack transcendental phenomenology as “Cartesianism”, as if its *ego cogito* were a premise or set of premises from which the rest of knowledge (…) was to be deduced, absolutely “secured.” The point is not to secure objectivity but to understand it (p. 193).

What is behind Husserl’s quite explicit rejection of understanding phenomenology as another form of “Cartesianism”? Although much can (and has been) said about this (see e.g., MacDonald, 2000; Staiti, 2015), in the present context, the most important point to stress is that Husserl’s and Descartes’ projects are driven by fundamentally different questions. The relevant difference for our purposes is to be found in the opposition Husserl draws in the passage above between the Cartesian project of ‘securing’ and that of phenomenological ‘understanding’. For Descartes, as is well known, given the rather unlikely but still imaginable possibility of my being deceived by a vicious demon, the crucial question becomes that of securing some ‘fundamentum inconcussum’ (involving the two figures of the cogito and a benevolent God) in order to ward off the skeptical threat of perpetual illusion. On a more formal level, Cartesian hyperbolic skepticism represents a passage from uncertainty regarding the truth of most of our convictions regarding the world and ourselves, to certainty with regard to the truth of believing itself (i.e., Koch’s thesis above). How then is this different from Husserl’s description of the task of phenomenology not to secure, but to *understand* objectivity? And how is this related to the issue of first-person incorrigibility and truth? Here is how Husserl (1989) specifies this task:

Its sole task and accomplishment is to clarify the sense of this world, precisely the sense in which everyone accepts it – and rightly so – as actually existing. That the world exists – that it is given as an existing universe in uninterrupted experience which is constantly fusing into universal concordance – is entirely beyond doubt. But it is quite another matter to understand this indubitability, which sustains life and positive science, and to clarify the ground of its legitimacy (p. 420).

So, on the one hand, Husserl would basically subscribe to Peirce’s (1992) sobering advice not to “pretend to doubt in philosophy what we do not doubt in our hearts” (1992, pp. 28–29), thereby denying that such a radical pretense is genuinely possible to begin with. Doubting, according to Husserl, is not a matter of simple decision or sheer will. In order to doubt something and to go beyond a philosophical play of mere make-believe, I need a relevant motivation to doubt and such motivation seems entirely lacking in the case of my experience of reality. The world, Husserl (1995) writes, stands before our eyes as existing “without question” (1995, p. 17); or, in Heidegger’s (1985) words, it is there “*before* all belief” and never “experienced as something which is believed any more than it is guaranteed by knowledge” (1985, p. 216). Basically all phenomenological authors would concur on this basic point: rather than being the effect of what I believe it to be, my experience of reality, in its own characteristic way of appearing, determines and makes possible any beliefs I might form about it in turn (see also Merleau-Ponty, 2002, p. 44 *et passim*).

Yet this, according to Husserl, still somehow constitutes a problem. Again, however, one should avoid precipitously identifying this ‘problem’ with the Cartesian effort of trying to secure the world or any of its objects through some kind of miraculous deduction starting from the immanent sphere of consciousness. The main reason for Husserl’s misgivings with regard to such an effort is not so much that it is a hopeless endeavor (although it is), but that it already betrays one’s lack of a proper understanding of consciousness and world-experience. That is, within this Cartesian setup, conscious experience is already pre-understood as a matter of having ‘subjective representations’ or ‘images’ that may or may not correspond to the object as it is in-itself standing behind the veil of appearances, thereby opening up the skeptical challenge of knowing whether and how knowledge can extend beyond the prison-house of subjective-relative appearances. This conception of consciousness as some kind of ‘bag’ or ‘container’ where representational contents, to use Dennett’s expression, “swim along” (1991, p. 44) is, in Husserl’s (1999) view, a “fatal mistake” (1999, p. 35).

To give just one example of the ‘mistake’ Husserl is aiming at: when I visually perceive, say, a chair, almost no one (except, perhaps, some philosophers) makes the mistake of claiming that what we are actually conscious of is some sort of image, sense-datum or another mental representative of the chair. According to Husserl, being conscious of the chair through the presentational consciousness of some other chair-like simulacrum precisely does not count as a perception of a chair, even if this representative image somehow resembles the original chair. This is (but one limited way) how our ordinary experience of everyday objects immediately strikes us, as somehow presenting us with the chair itself. Yet most of us, Husserl included, would agree that objects don’t appear before consciousness magically, as – in Sellars’ idiom – “a seal on melted wax” (Sellars, 1981, p. 12): at least some sort of process is involved that makes the presence of the object – in the way that it is present – possible. It is precisely this possibility which Husserl, under the heading of his transcendental-constitutive analysis, wants to *understand*: not *if* conscious experience is involved with the world itself rather than its ghostly double, but *how* it is so. Now the ‘fatal mistake’ – of which Descartes was in Husserl’s eyes a paradigmatic offender – occurs when we substitute some part of this process, i.e., that in virtue of which we are conscious of objects in the way that we do, for our consciousness of the object itself. In contemporary terms: when we confuse properties of *what is represented* with properties of the *representing*. To stick with the chair-example: as I visually perceive the chair, my experiences change in various detectable ways. What I perceive of the chair will, amongst other things, depend on my position relative to it. Yet, the chair itself does not appear to get bigger or smaller whenever I get nearer or back away from it, nor does it appear to turn black when I switch off the light. I perceive the chair as having one uniform color, size and shape, while the sensations in virtue of which I perceive those qualities can, and often must, vary with changes in my environment and condition. However, in straightforward perception I do not attend to these sensations, and they are not among the perceptual experience’s object. As Husserl (2001) writes: “I do not see color-sensations, but colored things, I do not hear tone-sensations but the singer’s song” (p. 99).

Although we won’t go further in Husserl’s detailed analyses of perceptual experience, Husserl’s notorious quarrel with Descartes’ theory of ideas on what it is like to perceive a world already reveals that there is nothing specifically ‘incorrigible’ about the phenomenological epoché and its turn to world-as-experienced. So unlike Koch’s reading of Descartes, the phenomenological version of the epoché does not entail any sort of insularity thesis according to which first-person beliefs are immune to error as long as they are restricted to the principle of appearance qua appearance. Put yet another way: not how I think the world appears, but how it actually appears is the principal focus of phenomenological inquiry. And this is precisely what the phenomenological epoché, on Husserl’s understanding, should enable us to do: no more and no less than to clarify the implicit sense of what is always already familiar and taken for granted in straightforward experience.

### Dennett’s Heterophenomenology: Cartesianism without a Theater

We seemed to have wandered needlessly into the historical vagaries of philosophical dispute between Husserl and Descartes in order to shed a light on an apparently trivial example like that of the phi-phenomenon discussed by Dennett. But, as pointed out endlessly by Dennett himself, seeming is not always being, and the triviality of the example stands in sharp contrast with the wider philosophical ramifications Dennett develops on a certain reading of the phenomenon for the very idea of consciousness and the first-person perspective. So what about this reading?

There is indeed a much simpler and parsimonious approach to the topic of subjective experience compared to the one we have been offering on our reading of Husserlian phenomenology. Dennett is a firm defender of the methodological principle that scientific theories, especially when it comes to such a contested field as consciousness, should avoid multiplying entities beyond necessity (1991, p. 134). By this he means primarily that if a certain gap should arise between how things stand in reality and how they ‘seem’ from the first-person perspective, one should avoid postulating what he famously calls ‘the Cartesian theater’: i.e., some kind of inner screen where shadowy representatives of an absent reality are henceforth *really* present before the inner eye of an equally shadowy observer. Yet, as Dennett points out, the idea dies hard and in order to dispel its seductive lure, it is not enough to avoid explicit reference to outdated forms of dualistic metaphysics by, for example, quickly replacing mind-talk with brain-talk. That is, even within the now dominant terms of neurocognitive materialism, the Cartesian specter tends to make its unholy reappearance – under the bastard idea of ‘Cartesian materialism’ – whenever a central point is presupposed where everything “comes together” and various brain processes present their final product to consciousness (Dennett and Kinsbourne, 1992, p. 185). Such an idea is pernicious in a variety of ways: on the one hand, it usually makes for a sloppy functional analysis of how the brain is able to perform its various capacities because such an abstract center tends to clean up all our functional loose ends. On the other hand, wherever there is talk of something being finally presented to consciousness, we can be sure that the idea of a central observer appreciating and observing this product is never too far away. As Dennett (2007) points out with regard to the example visual consciousness:

We are making steady progress on this subpersonal story […] and we can be confident that there *will be* a subpersonal story that gets all the way from eyeballs to reports and in that story there *will not be* a second presentation process with an inner witness observing an inner screen and then composing a report. As I never tire of saying, the work done by the imagined homunculus has to be broken up and distributed around (in space and time) to lesser agencies in the brain (p. 11).

This is the crux of Dennett’s Multiple Drafts Model, specifically devised as an antidote to the crippling but tenacious legacy of the Cartesian Theater. However, what exactly does it mean to deny the existence of this central ‘Oval Office’ and to replace it it with “various events of content fixation occurring in various places at various times in the brain” (Dennett, 1991, p. 365) for our overall discussion on first-person appearance? Did we, for example, not already see how Husserlian phenomenology left behind such a theater-conception of consciousness quite some time ago for an externalist understanding of consciousness as essentially world-involving? To appreciate in what way Dennett’s proposal specifically diverges from the phenomenological tradition, let us see how this model works *in vivo* with the empirical example of the phi-phenomenon.

In response to Goodman’s question (cf. supra) for a possible explanation of the avowed experience of seeing the moving spot change color before it actually occurred, Dennett considers two possible hypotheses: an Orwellian and Stalinesque version, respectively. That is, either we concoct false memories of *having seen* the spot change color, though in fact we had no prior immediate experience of the change (this is the post-experiential ‘Orwellian’ revision, suggesting a kind of *ex post facto* rewriting of history); or else we really do have a genuine, albeit delayed, consciousness of the light moving continuously and changing color, due to a kind of unconscious rapid editing process that “fills in” the missing data and presents the finished product to the eye of the beholder (this is the pre-experiential ‘Stalinesque’ revision, reminiscent of the cooked-up evidence presented in communist show trials, which does in fact make a genuine appearance in court, despite its fraudulence). Common to both versions is the subject’s *belief* in the conscious experience of the moving spot, although they seem to differ substantially in how that belief came about: due to false memories of a veridical experience in the Orwellian case, and accurate memories of a non-veridical experience in the case of the Stalinesque show trial.

Yet, in glossing over these two accounts of the phi-phenomenon, Dennett’s ‘counterintuitive’ proposal finally reveals its true color: according to Dennett, the intuition that one of the explanations must be correct to the exclusion of the other is, once again, a clear indication of our persistent captivity in the Cartesian Theater. Postulating a difference between *a real experience* above and beyond or, more properly, before or after what subjects *believe they experienced* is, in Dennett’s functionalist idiom, “a difference that makes no difference” (1991, p. 132). Hence, in clear opposition to the phenomenological account, the conclusion Dennett wants us to draw from the numerous experiments discussed in his *Consciousness Explained* is that there is no difference between how things *seem* to us and how we *think* they seem. Bringing this all together, Dennett consequently dubs his Multiple Drafts Model as a *first-person operationalism* “for it brusquely denies the possibility in principle of consciousness of a stimulus in the absence of the subject’s belief in that consciousness” (1991, p. 132).

Needless to say, such a conception of the first-person perspective as entirely constituted by subjective beliefs immediately carries some remarkable consequences for the scientific effort to devise a proper naturalistic account for this dimension. These become most salient in Dennett’s own methodology of choice for approaching the scientific study of human consciousness, his *heterophenomenology*, advertised as an explicitly *third*-person approach to the issue of subjectivity (Dennett, 2003). On the face of it, this seems like a remarkable thing to do: subjectivity is traditionally regarded as occupying something rather like another dimension – the private, ‘first-person’, more or less self-consciousness sort of being – in comparison with the privileged objects of ‘third-person’ methodologies like meteors, sound waves or bone density. This ostensible difference between meteors and minds has led some phenomenological authors, like e.g., Evan Thompson, to claim that phenomenology, as an explicitly first-person investigation, is in fact an inescapable prerogative for any naturalistic account because “any attempt to gain a comprehensive understanding of the human mind must at some point confront consciousness and subjectivity – how thinking, perceiving, acting and feeling are *experienced in one’s own case*” (Thompson, 2007, p. 16, our italics; see also Gallagher, 2007). The reasoning behind such a claim is relatively straightforward and is meant particularly as a warning sign for Dennett’s – but also others’ – overambitious naturalistic inclinations: any explanatory scheme which departs so completely from first-person experience that it calls into doubt the very existence of such experiences can no longer claim to be elucidating that explanandum, since it has effectively ‘eliminated’ the very object it purports to be an account of. Yet Dennett is not impressed. In an essay addressing some misunderstandings concerning his apologia for his heterophenomenology with the little concealing title “Who’s On First?”, Dennett asks:

Can the standard methods be extended in such a way as to do justice to the phenomena of human consciousness? Or do we have to find some quite radical or revolutionary alternative science? I have defended the hypothesis that there is a straightforward, conservative extension of objective science that handsomely covers the ground – *all* the ground – of human consciousness, doing justice to all the data without ever having to abandon the rules and constraints of the experimental method that have worked so well in the rest of science (2003, p. 19).

Heterophenomenology is the method of choice whenever one wishes to take the first person point of view seriously – that is, as seriously as it *can* be taken – while keeping the methodologically scrupulous requirements undergirding the same objective perspective that reigns in those sciences that aspire to explain different, but from a methodological point of view, no more daunting phenomena such as continental drift, biodiversity or the digestive tract. So what then, exactly, does Dennett’s heterophenomenology commit us to? What precisely does it mean to take subjects seriously, but not too seriously?

### Return of the Cogito: Serious, All Too Serious

Given that we have to adopt a strict third-person perspective, since as Dennett is keen to say, “*all* science is constructed from that perspective” (1991, p. 68), the only access to the phenomenological realm will be via the observation and interpretation of publicly observable data, including utterances and other behavioral manifestations. Furthermore, we will have to find a neutral way of describing these data – that is, a way that doesn’t prejudge the issue – by adopting a special form of heterophenomenological abstinence. Such an abstinence – likened by Dennett to a third-person version of Husserl’s epoché (Dennett, 2003, p. 22) – brackets the issue about whether the subject is in fact a computer, a mere zombie, a dressed up parrot or a real conscious being. The only thing such a third-person epoché *does* commit us to is adopting what Dennett calls *the intentional stance*: we should interpret the vocal sounds emanating from the subjects’ mouths as speech acts that express the subject’s beliefs in order to compose a text of what that subject wants to say about his or her own conscious experiences. As Dennett (1991) explains:

People undoubtedly do believe they have mental images, pain, perceptual experiences and all the rest, and these facts – the facts about what people believe, and report when they express their beliefs – are phenomena any scientific theory of mind must account for. We organize our data regarding these phenomena into theorists’ fictions, “intentional objects” in heterophenomenological worlds. Then the question of whether items thus portrayed exist as real objects, events, and states in the brain – or in the soul, for that matter – is an empirical matter to investigate (p. 98).

Again, this merely seems like a fairly trivial rehearsal of the principle of scientific neutrality: from the fact that people believe all kinds of things, even when these things happen to refer to their own experience, we shouldn’t immediately conclude that these things actually exist. As a case in point, take the example of visual experience. People often seem to believe that their visual experience represents the world in sharp focus, uniform detail and high resolution from the center out to the periphery, as a kind of snap-shot like picture of the world (Noë, 2004). Yet as Dennett points out, convincingly, such a view of visual experience as gap-free and wholly continuous is mistaken. Take the example of looking at a wall: while staring at it, it looks to you as if there is unbroken expanse of wall. However, this is not to say that it seems to you as if, in a single saccadic fixation, the whole of the wall’s surface is presented in equally rich detail from the center out to the periphery, as if every part of the wall’s surface is immediately presented to consciousness at once. Rather, as Husserl would say, the wall is “transcendent” to my experience insofar as it has more to it than what is revealed to me in any single phase of experience and, moreover, is precisely *given* or *experienced* (as opposed to inferred or deduced) *a*s having more to it. So, as the example illustrates, we would do well to follow Dennett’s advice by not taking everything a subject believes about his or her own experience at face value. On this account, taking subjects’ reports and beliefs serious means that we take into account that they can be mistaken; if not, that is, if doubt with regard to conscious experience would be strictly speaking senseless, then – as Wittgenstein (1958) famously said – so too would any assertion of knowledge (1958, p. 211).

However, despite Dennett’s repeated insistence on our pervasive fallibility with regard to first-person experience as a central argument for the adoption of his heterophenomenological stance, the question is how this methodological modesty squares with his aforementioned first-person operationalism. How to reconcile the by all parties acknowledged fact that we can be wrong about first-order experience with Dennett’s thoroughgoing fictionalization of subjectivity up to the point where we have “total, dictatorial authority over the account of how it seems to you, about what it is like to be you” (Dennett, 1991, p 96)? To put it in the language of Dennett’s own cherished functionalism: is fallibility a difference that can make a difference for the defense of heterophenomenology when there is no possibility of consciousness in the absence of a subject’s belief in that consciousness? It must be clear by now that it cannot, and furthermore, that this inability to make room for a distinction between appearance and reality within the field of conscious appearance is itself a remarkable and, as per the defense of heterophenomenology, undesirable consequence of what we would call Dennett’s own doxastic Cartesianism. For if my subjective experience is entirely constituted by what I believe to be the case, or what I believe that seems to be the case, then there is of course – and as we made clear in our reading of Descartes – no ‘case’ about which I can be wrong either. Consequently, Dennett is confronted with the following paradox:

(1) Either make room for the distinction between experience and judgment in which case the plea for heterophenomenology can be saved but at the cost of first-person operationalism;
(2) Or retain the latter, but at the cost of being unable to countenance fallibility and hence of introducing an unavoidable return to the philosophical core of Cartesianism, although this time without a theater.

Yet secondly, apart from this general philosophical tension, a further problem for Dennett’s doxastic account arises when we raise the further question about how it construes the relation between belief and experience. As we have seen, the central strategy of Dennett’s heterophenomenology is to attribute a belief to the subject whenever he or she reports an experience, rather than the other way around. And indeed, it has been a traditional argument ever since Descartes and Kant that an ‘I think’ or ‘I believe’ accompanies, or at least in principle *must be able* to accompany, any experience I might claim to be having, for otherwise, as the famous quote from Kant (1996) continues, “something would be represented in me that could not be thought at all, which is as much as to say that the representation would either be impossible or else at least would be nothing to me” (1996, B131–132). Importantly, Dennett trades on this ineliminable occurrence of the ‘I think’ within reflective consciousness to advance his first-person operationalism and thus to deny the possibility that there could be any difference between beliefs and experience. Since, as Dennett’s argument goes, if you have conscious experiences you don’t believe you have – those experiences would be just as inaccessible to you as to the heterophenomenological observer. Conversely, if you believe you have conscious experiences you don’t in fact have, it is your beliefs that need to be explained, not the non-existent experiences (Dennett, 2003, p. 21). Hence, the crucial question becomes: is it really necessary for a subject while enjoying or undergoing an experience, to immediately believe that he is enjoying or undergoing that experience?

It is safe to say that one of the grounding motives in the phenomenological works of most notably Sartre, Merleau-Ponty and Heidegger is a forceful critique of this intellectualistic idea. The claim has been that there are indeed conscious intentional experiences that a subject could have without immediately believing that he is having them. Even more, those experiences precisely depend on the subject’s *not* having a belief about them: if the subject were to believe he was having the experience, instead of merely living it through, the experience would become impossible (see also Dreyfus and Kelly, 2007). Differently put: a necessary condition for those types of experience is precisely the *absence* of the Cartesian spectator. A famous example is of course the following one by Sartre (2004):

When I run after a streetcar, when I look at the time, when I am absorbed in contemplating a portrait, there is no I. There is consciousness *of the streetcar-needing-to-be-overtaken*, etc., and non-positional consciousness of consciousness. I am then plunged into the world of objects; they constitute the unity of my consciousness, they present themselves with values, with attractive and repellant qualities – but me, I have disappeared (2004, p. 32).

Central to such examples is that the subject, to put it in Lacanian terms, is ‘internally excluded’ from the experience while living them through: in keeping an appropriate distance while talking to people, in reaching automatically for the proffered handshake, laughing while listening to some amusing story, rapidly walking down the stairs or backing away from a painting to get a better view, there is a certain solicitation from the world to engage in various activities without us having to decide to do so. Moreover, reflexively deciding, contemplating or believing in such activities is the best way to lose all grips on what the situation calls for. Such an experience of spontaneous immersion or absorption “into the world of objects” only arises precisely when we are not looking for them; conversely, they would vanish immediately insofar I would try to think or believe them. Yet, *pace* Dennett, this does not mean that in those instances I momentarily become a zombie or unconscious machine; there still is something ‘it is like’ to be absorbed in these activities which set them apart from, say, a comatose condition, and the absence of belief is one example of its constitutive features. It is precisely this ‘internally excluded’ character of belief and experience in such phenomena which Dennett’s doxastic Cartesianism cannot countenance.

## Dennett and Psychoanalysis: The Curious Case of the “Objectively Subjective”

Before we can begin to specify in what way psychoanalytic theory could bear on this debate between Dennett and phenomenology, some precursory remarks are in order. Because, first of all, it might not be entirely clear where, if at all, we should situate psychoanalysis, whether in its original Freudian formulation or in its Lacanian version, along Sellars’ somewhat contrived, but perhaps still hermeneutically useful dichotomy. For in contrast to phenomenology’s straightforward and programmatic insistence on first-person experience as an indispensable datum for any sort of naturalism, the claims and subject matter of psychoanalytic theory are in this regard, to put it mildly, somewhat more ambiguous. Of course, it might be argued that psychoanalysis is not really that interested in, nor in itself capable of, pursuing or resisting the naturalistic agenda of bridging the gap between neurocognitive and phenomenological accounts of blind sight or the rubber-hand illusion. And indeed, one could say, what would be further removed from psychoanalysis’ basic insight into man’s constitutive *disadapted* relation to its ‘natural’ environment than Dennett’s talk of the design stance? Yet such a first and perhaps heartening impression is easily discarded when we shift the terrain to those phenomena, however, ill designed, that are considered to be at least more amenable to psychoanalytic elucidation, such as the nature of psychopathology, sexuality, language or desire. With regard to those, the question emerges whether or not psychoanalysis would simply join in with Dennett’s deflationary tactics of reducing the several issues that may arise in the effort of articulating the intertwinement of subjectivity and language – for example, the relation a subject entertains with its own speech – or subjectivity and desire – for example, what it means to desire the desire of the other – to the austere categories of ‘seeming’ or ‘fiction’? Presumably, the pre-reflexive answer would consist in a quick and resounding ‘no’.

However, on the other hand, given Freud’s departure from Brentano’s views on intentionality and self-consciousness (see Seron, 2015, pp. 15–30; Bernet, 2002) or Lacan’s critique of both Sartre’s existential psychoanalysis (Lacan, 2006, p. 80) and Merleau-Ponty’s bodily unconscious (Lacan, 1982-1983, p. 75), it is equally clear that the intellectual relation between phenomenology and psychoanalysis is complicated enough to avoid any easy wedding of psychoanalysis with manifest experience. Given this ambiguous position, a clarification of how psychoanalysis relates to the ongoing ‘scientific disenchantment’ along the terms set by Dennett’s ingenious codification of the claims and methods of cognitive science, and conversely, to the phenomenological arguments that are being advanced against Dennett’s proposal, indeed becomes a reasonable task.

### Freud and Lacan between Phenomenology and Cognitive Science

To begin with some sweeping examples: what exactly is left of the once so seductive Freudian image of the three successive humiliations of man-in-the-world (Copernicus-Darwin-Freud) now that we can safely add a fourth one, -Dennett? As noted by several commentators (Gardner, 2000; Zizek, 2006), it seems that Freudian psychoanalysis, steeped as it is in the intentional language of repression, desires and negation – now finds itself amongst those traditional ‘manifest’ philosophies threatened by the more recent neurocognitive humiliations. And the reason for this clear: though, in the felicitous wordings of Freud, “psyche is extended; knows nothing about it” (Freud, 1938b, p. 300), there has never been a lack of critics pointing out that what it does not know is still suspiciously close to the philosophical problematic of intentional consciousness. And it is indeed striking and ironic, given psychoanalysis’ original declaration of independence from any philosophy issuing from the cogito (see Freud, 1923, p. 13; Lacan, 2006, p. 75), that one of the standard ways of defending the unconscious today is precisely by referring to the properties of consciousness, of which intentionality is held to be pre-eminent. As Freud (1915) himself indeed pointed out:

All the categories which we employ to describe conscious mental acts, such as ideas, purposes, resolutions, and so on, can be applied [to unconscious mental acts]. Indeed, we are obliged to say of some of these latent states that the only respect in which they differ from conscious ones is precisely in the absence of consciousness (p. 168).

Turning to Lacan, we can discern the same manifest motives despite the latter’s proto-cognitivist, structuralistic inclinations: even on the most cursory reading of the *Ecrits*, it would be difficult to avoid the perennial ‘philosophemes’ of subject, alienation, desire, Other and so on. But Lacan, of course, would try to avoid any quick philosophical (mis)recognition by reminding his readers to this subject is split, barred and divided by the signifier, therefore not the phenomenological subject of *Jemeinigkeit*, always already conscious of itself in its intentional involvement with the world. Nevertheless, however, split or deferred this subject might be, this failed or misfired cogito is still close enough to its Cartesian predecessor so as not to completely break with it. On the contrary, Lacan only ever wanted to think the unconscious, not as antithetical to consciousness, not as “a species that defines a circle in psychical reality of what does not have the attribute of consciousness” (Lacan, 2006, p. 830), but precisely as the implicit truth already contained in the Cartesian *cogito me cogitare* (see Dolar, 1998; Lacan, 2006). But this also means that this truth, whatever else it may be, is the truth *of* that same old Cogito, gained through a certain radicalization of that traditional aporia, confronted differently by Husserl, Fichte or Sartre, of conceiving subjectivity from and within its own terms. Therefore, in so far as phenomenology is susceptible to Dennett’s heterophenomenological recasting of subjectivity in terms of a ‘theorist’s fiction’, the same holds for Lacan’s particular defense of the subject. And is it indeed not quite imaginable, for example, that Dennett’s response to Lacan’s notorious anti-humanistic formula in *Science and Truth* according to which “science’s man does not exist, only it’s subject does” (2006, p. 730) would be the sobering “nor does it’s subject, although it might *seem* to”?

On the other hand, however, it could be pointed out that we might have proceeded a little bit too quickly and carelessly in lumping together psychoanalysis with the manifest philosophies of intentional experience. And in fact, as is well known, both Freud and Lacan have been taken to task for neglecting and underestimating the accomplishments of phenomenology in a way that is, although not strictly equivalent, remarkably close to the contemporary phenomenological arguments raised against Dennett (see Schafer, 1976; Chalmers, 1995). Suffice to recall, in Freud’s case, the succinct phenomenological critique of the psychoanalytic unconscious by Eugen Fink in the appendix to Husserl’s *Crisis*:

This objection, which is raised in many variations against the so-called ‘consciousness-idealism of phenomenology,’ is based on a fundamental philosophical *naïveté*. […] The *naïveté* we refer to consists, before all theory-construction about the unconscious, in an *omission*. One thinks one is already acquainted with what the ‘conscious’, or consciousness’, is and dismisses the task of first making into a prior subject matter the concept against which any science of the unconscious must demarcate its subject matter, i.e., precisely that of consciousness. But because one does not know what consciousness is, one misses in principle the point of departure of a science of the ‘unconscious’ (Husserl, 1970, p. 386).

Despite the shifting terminology, it’s not too difficult to recognize in this depiction of psychoanalysis’ ‘naïveté’ the same point of contention we already encountered in Dennett’s case. That is, Freud is held to have tried to develop a naturalistic third-personal theory of mental functioning, couched in broadly functionalistic-energetic terms (the dynamic trinity of ‘ego’, ‘id’ and ‘superego’; the energetic metaphors of cathexis, displacement, condensation, and so on), destined to explain various pathological phenomena, without, however, offering any explicit and sustained consideration of ‘consciousness’. As noted by Tugendhat (1986, pp. 131–132) and Ricoeur (1977, p. 420 *et passim.*), Freud indeed never seems to talk about phenomenal consciousness and self-consciousness directly, but only and rather loosely in terms of the ‘ego’ which is portrayed as yet another homuncular-functional entity amongst others. A similar story can be told with regard to the notion of repression and other ‘mechanisms’ of defense of which Freud gives an extended account in terms of word- and thing-presentations, cathexes and so on, again strongly suggesting that these concepts designate sub-personal operations of some kind. Accordingly, repression, displacement, condensation and other similar notions, should not be conceived as first-personal actions, whether conscious or unconscious, operating within a normative framework of beliefs and desires, but as functionally specifiable processes requiring no agents, experiencers or observers whatsoever. In short: on this reading, psychoanalytic theory turns out to share roughly the same agenda as cognitive neuroscience in trying to offer a homuncular-functionalist top-down analysis of mental functioning in terms of various subsystems until we reach the level of non-intelligent functionaries that can be replaced by a machine (see Freud, 1938a, p. 144).

As for Lacan, although one of the remarkable features of his self-proclaimed return to Freudian orthodoxy is precisely the explicit avoidance of all those orthodox-naturalistic elements, this didn’t necessarily seem to imply a return to a consideration of *l’expérience vécue* at the expense of Freud’s naturalism. On the contrary, Lacan’s structuralist-materialist reworking of Freudian metapsychology in terms of his ‘logic of the signifier’ appears to have been driven by an even more fierce ambition to finally establish psychoanalysis as a rigorous, because aspiring to the Koyréan ideal of mathematical formalization, scientific endeavor (see Milner, 1995; Johnston, 2012; Tomšic,, 2012). And indeed, after some years of flirting with phenomenological accounts, the famous 1953 *Discourse of Rome* can be seen as a veritable trumpet blast fiercely announcing the new footings on which to resurrect the psychoanalytic edifice as a “science of subjectivity” (Lacan, 2006, p. 235):

The form of mathematicization in which the discovery of the *phoneme* is inscribed, as a function of pairs of oppositions formed by the smallest graspable discriminative semantic elements, leads us to the very foundations that Freud’s final doctrine designates as the subjective sources of the symbolic function in a vocalic connotation of presence and absence. And the reduction of any language (*langue*) to a group comprised of a very small number of such phonemic oppositions, initiating an equally rigorous formalization of its highest-level morphemes, puts within our reach a strict approach to our own field (Lacan, 2006, pp. 235–236).

This is indeed a far cry from Lacan’s earlier phenomenological declaration that “language, prior to signifying something, signifies to someone” (2006, p. 66). Here we are incited to the view that language, loosely defined in terms of the symbolic order as somehow pertaining to both the regulatory principles of social exchange and the material-phonetic level of ‘pure signifiers’ (on this equivocation, see Descombes, 2009), is “a form in which the subject is inserted at the level of his being” (Lacan, 1993, p. 179). In the *Seminar on “The Purloined Letter*” and *The Instance of the Letter*, gloomy images are being invoked to convey the radical nature of this ‘insertion’. Lacan (2006, p. 21)) talks about “machines-that-think-like-men, subjects “more docile than sheep”, lined up before the gripping effect of the signifying chain as “ostriches”, determined by the signifier’s displacement in their “acts, destiny, refusals, blindnesses, success, and fate.” How all of this is supposed to be an effect of ‘symbolic determination’ is never really convincingly spelled out (Dennett’s anti-mysterian concept of “wonder tissue” might be put to good use here; see Dennett, 2013, pp. 198–205), nor is it clear if and in what way Lacan’s structuralistic remolding of Freudian concepts (e.g., metaphor/metonymy for condensation/displacement, repetition of the signifying chain for *Wiederholungszwang*, …) is really an advance compared to the latter. Yet, for our present purposes, at least it is clear that on this reading Lacan’s intent was to purify the Freudian unconscious even further from any remaining residues of metaphysical humanism and its associated anthropomorphic terminology. Hence, as we have noted in the case of Freud, Lacan’s structuralistic rearticulation of the unconscious could therefore equally well be seen as a forerunner to the sort of materialist program better known today under the heading of cognitive science (see also Dupuy, 2000).

### Psychoanalysis’ “Abominable Mess”

Now this Janus-faced character of psychoanalysis has of course never slipped the attention of its various commentators, provoking blame and rejection from its critics; praise, confusion or silent endorsement from its defenders. On Sartre’s diagnosis, for example, psychoanalysis is precisely founded on a systematic confusion of the personal and sub-personal levels and this constitutes its unavoidable yet inadmissible paradox: “I both *am* and *am not* the psychic facts hypothesized by psychoanalysis” (Sartre, 1958, pp. 50–51). In roughly the same way, Wittgenstein famously spoke of psychoanalysis’ “abominable mess” (Wittgenstein, 1978, p. 316), premised as it is on a confusion between first-personal reasons and third-personal causes, imposing the former even if the patient doesn’t accept them, resorting to the latter and being content with the patient’s acceptance for their justification (Cioffi, 1998).

Similar accusations apply to Lacan’s ‘materialism of the signifier’, as representing yet another confused effort to straddle both sides of this fence at once: the signifier being regarded as both amenable to a ‘materialist’ description in terms of its meaningless phonemic properties and an ‘idealist’ description in terms of its meaningful semantic properties. By cutting the cord between signifier and signified, and in the same move declaring the primacy of the former over the latter, Lacan thought to be able to transmute these ‘pure signifiers’ into causal forces operating in a mechanical way, yet responsible for the different meaning effects encountered in psychoanalytic practice. This is, for some, the very formula of magic (see Descombes, 2001, p. 93–101).

So what then are we to make of this confusion? There are of course some well-tried reconciliatory attempts which basically consist in either translating psychoanalysis into phenomenological terms^2^, or, embracing it precisely as a form of sub-personal psychology^3^ (see Gardner, 2000 for a detailed discussion), yet perhaps the more interesting thing to notice is the recurrent image from which such solutions spontaneously spring. By this we mean, as regards the present discussion, the very form in which the difference between Dennett and phenomenology is formulated: that is, in terms of ‘fictional beliefs’ (Dennett) and ‘manifest experience’ (phenomenology). In the following section, we will therefore try to explicate in what way psychoanalytic theory could distinguish itself from both these readings.

### Decentering Appearance: Belief in Belief

Let us begin this section by recapitulating in a condensed form the core disagreement which stands behind the discussion between Dennett and phenomenology. Whether it is the topic of perceptual consciousness, imagination, sense of agency, the painfulness of pain or the problem of other minds, is not really that essential here: the crucial divergence already appears at the level of “the organization of the data” (Dennett, 2003, p. 9), that is, the way in which researchers should proceed whenever they set out to explain those phenomena of consciousness.

In his *Who’s on First?* (2003), Dennett offers a clear summary of his position that can put the problem in sharper focus. “What”, Dennett asks, are the “primary data” from which we ought to depart in order to arrive at “a science of consciousness” (p. 21), i.e.: (a) conscious experiences themselves; (b) beliefs about these experiences; (c) verbal judgments expressing those beliefs; or (d) utterances of one sort or another? Whereas, as we have seen, phenomenology vigorously opts for (a); Dennett’s heterophenomenology is premised on the elimination of (a) in favor of (b) and (c). From this disagreement, it necessarily follows that the philosophical concepts of ‘appearance’ and ‘seeming’ function as a kind of *shibboleth* in the ensuing debate: designating ‘immediate experience’ in the case of phenomenology, ‘beliefs about experience’ in Dennett’s account. Furthermore, it becomes obvious why the way in which arguments are being pressed for one position or the other, take on the form in which we have argued for some of the claims found in phenomenology: i.e., those cases that point toward a *disjunction* between belief and experience – that is, on the one hand, ‘beliefs without experience’ as in the discussion about the level of detail found in visual consciousness; on the other hand, ‘experience without belief’ as in Sartre’s example of ‘pre-reflexive immersion’.

From a psychoanalytic perspective, however, what should immediately strike us is not so much this manifest level of disagreement in terms of the difference between ‘belief’ and ‘experience’, but rather the implicit Cartesian agreement between Dennett and phenomenology on the concept of *belief* itself. To put it in Spinozistic terms, there is still, however, slightly, something sticking out as ‘a kingdom within a kingdom’ throughout this whole discussion, something which becomes most explicit in Dennett’s response to some of his critics – Schwitzgebel (2007) and Noë (2007) – in his *Heterophenomenology Reconsidered* (2007). As before, the critique involves Dennett’s seemingly incompatible insistence on both the essential fallibility of first-person accounts and some kind of Rortian incorrigibility with regard to “how it seems to them” (p. 263). In the same volume, Noë (2007) evokes the familiar Wittgensteinian theme on the logical limits of justification of first-person accounts which comes to an end rather sooner than we might think:

It [Wittgenstein’s view on justification] applies to the special case where what is in question is how I take my own experience to be, what I take it to be like. I *can* be mistaken about the nature of my experience – about how I, in experience, take things to be. […] But it would be a different kind of mistake for me also to be mistaken about how I *take* my experience to be. I can be wrong then about how things seem but not wrong about how I take things to seem (p. 244).

Dennett (2007) wholeheartedly agrees:

If somebody says her visual field *seems* detailed all the way out to the periphery, which lacks a perceptible boundary, there is no gainsaying her claim, but if she goes on to theorize about “the background” […], she becomes an entirely fallible theorist, no longer to be taken at her word (p. 263).

So, at least when it comes to this notion of ‘taking’, of what I believe my experience ‘is like’, phenomenology and Dennett prove themselves to be something like an “epistemological pair” (Bachelard, 1949, p. 5). In short: whereas we can be wrong about ‘seeming’ understood in terms of experience, we cannot, however, be wrong about ‘seeming’ in terms of belief. Now the proper psychoanalytic questions that should arise in this context are the following ones: whence this sudden confidence? Which arguments are being advanced here to uphold this last inviolable sanctum of minimal infallibility with regard to this Cartesian theme of ‘belief in belief’? Why is it so obvious that ‘immunity to error’ immediately ensues whenever we shift the terrain to the topic of belief itself? Is it because, in this case, the well-known phenomenological figure of the ‘unbeteiligte Zuschauer’ suddenly reappears on the scene? If so, why would that be? And why precisely at this point? Surprisingly, precious little can be found on these questions when we consult these various commentators.

Nevertheless, here, as everywhere else, it could be wise to stick to Althusser’s famous “golden rule of materialism” to “not judge a being by its self-consciousness (Althusser, 1996, p. 115). Particularly interesting in this context are the analyses we can find in the works of Zizek (1998, 2006, 2014) and Pfaller (2014, 2015) on the psychoanalytic concept of ‘belief’. Elaborating on and extending Octave Mannoni’s perspicuous theory of fetishism (see Mannoni, 2003, pp. 68–92), both these authors have drawn attention to a series of entirely common phenomena which nevertheless share some striking features that are directly relevant for our overall discussion. To start with some of these examples:

Imagine you’re sitting in a bar reading a newspaper, waiting for a friend. The friend arrives. He says hello, and then continues: ‘Excuse me, can I have a quick look at your newspaper? I know it’s silly, but I just have to know the score from yesterday’s game’ (Pfaller, 2014, p. 1).

For reasons that are no doubt suspect, but hidden, I sometimes read the rather rudimentary horoscopes published in certain papers. It seems to me that I do not take much of an interest in them. I wonder how people can believe in them. […] Once, last year, my horoscope said that “tomorrow will be an extremely favorable day for tidying up the house.” This was not a spectacular prediction, except that I had long been planning to move on the day in question. I burst out laughing at so funny a coincidence. […] I could say that I am not superstitious because I pay no mind to such things. But, to be precise, I should rather say: I know quite well that coincidences of this kind are meaningless, but I take a certain amount of pleasure in them all the same (Mannoni, 2003, p. 78).

What is common to these examples – and numerous others (see Zizek (1998); Pfaller (2014), for an extensive collection) – is the paradoxical relation a subject seems to entertain with its belief (i.e., the importance of sports; the significance of horoscope predictions). In a way that is difficult to render conceptually transparent, these sorts of beliefs are never really believed in (“I know it’s silly” – “I know quite well that these coincidences are meaningless”), yet in one way or another, they nonetheless exert a particular influence on their subjects (the compulsion to look at the paper – the pleasurable laughter). Moreover, as Pfaller argues, it is not so much their *content* that is primarily of interest here, but rather the *form* in which people refer to these beliefs. That is, as was already apparent from Mannoni’s formula for these types of beliefs (“I know quite well … but still”), its form is characterized by a complex coexistence of ‘better knowledge’ and ‘belief’. The fidget sports fanatic knows quite well that yesterday’s results are not important, but still he *has* to see them. Despite the better knowledge, and despite the gap this places between him and his silly practice, he nevertheless acts *as if* sports are of utmost importance. Similarly, horoscope predictions seem rather ridiculous to Mannoni, nonetheless, as he admits: “if the horoscope had said that “tomorrow will be an extremely unfavorable day for moving”, “it would have made me laugh differently” (2003, p. 78.).

So in an initial approximation of the formal structure of this phenomenon, one should say that, on the one hand, these are beliefs that are never really one’s own beliefs in the sense that one would claim ownership over them (à la “I believe in horoscopes, I really do”). The “better knowledge” is immediately invoked to signal such a subjective position of transcendence toward the belief. Yet, different from, for example, the Sartrean formula for transcendence – i.e., “To believe is to know that one believes, and to know that one believes is no longer to believe” (Sartre, 1958, p. 114) – such a better knowledge seems to be of little avail with respect to the efficacy of the belief in question. On the contrary, as Mannoni points out, one should even wonder if this position of knowledgeable transcendence does not somehow *contribute* to the maintenance of the disavowed belief? Such a claim would immediately contradict the seemingly incontestable principle according to which the dialectical relation between ‘knowledge’ and ‘belief’ is grounded in *exclusion*: on such an account, we (erroneously) believe (in the importance of sports, in the predictive validity of horoscopes, …) precisely because we do not know (that sports are not important, that horoscopes do not possess predictive value, …); conversely, better knowledge cancels out what we previously believed. Here, on the contrary, the dialectical relation between knowledge and belief would be based on the principle of *necessary conjunction*: only on the condition of better knowledge are we susceptible to those beliefs; conversely, in the absence of better knowledge, believing becomes impossible – as Mannoni succinctly puts it: “Evidently, the sole reason for the “but all the same” is the “I know well”” (2003, p. 71). If this principle is somehow sustainable (cf. infra for further argumentation), we can now proceed to advance a more precise formulation of this dialectic in terms of our general discussion: on a psychoanalytic account, the way things appear to me, that is, of what I “objectively believe” (cf. Zizek, 1989, p. 35) – as evidenced, despite myself, in my nonsensical behavior, spontaneous reactions and utterances (e.g., encouraging my car by means of little compliments when it won’t start, wearing my ‘lucky shirt’ for an important interview which I did prepare for in a ‘professional’ way, …) – as opposed to what I believe to believe, is structurally dependent on the self-reflexive movement of intellectual distancing through ‘knowing that one knows’.

### Belief in the Uncanny or Uncanny Belief?

Now, in order to substantiate this rather strong claim, what we seem to need is a methodological procedure closely resembling the phenomenological tool of “eidetic variation” (see Husserl, 1977, sec. 9a) in and through which, formally speaking, the necessary conditions of a certain phenomenon are established by ‘varying’ those conditions up to the point where the phenomenon itself would become (in)conceivable. Now it happens that a procedure of this kind is to be found implicitly in Freud’s 1919 treatise on *The Uncanny*, a phenomenon which, moreover, bears some striking resemblances to the examples discussed above (see Dolar, 1991; Zupancic, 2005; Pfaller, 2006). Regardless of the wealth of literary examples and psychoanalytic concepts Freud employs throughout the article, this dense and, to a certain extent, inconclusive text, essentially revolves around one central effort: to circumscribe the “common core” which would allow us “to distinguish as ‘uncanny’ certain things which lie within the field of what is frightening” (Freud, 1919, p. 219). Freud’s question is: how should one proceed in defining the common denominator in such a variety of examples as the mechanical doll Olympia, the obscene figure of the Sand-Man, the recurrent theme of the “double”, the “repetition factor” or the Rat Man’s “omnipotence of thoughts” – all taken as typical instances of the uncanny (Freud, 1919)? Why are they not simple examples of garden variety phobias such as the fear of heights or crowded places? What is it that sets them apart from the more epistemologically innocent logic of cognitive surprise or unfulfilled anticipation? And, finally, why should they even produce this peculiar affective response rather than none at all? As an instructive introduction for a further demarcation of the problem, Freud offers the following, general formula:

[…] an uncanny effect is often and easily produced when the distinction between imagination and reality is effaced, as when something that we have hitherto regarded as imaginary appears before us in reality […] (1919, p. 242).

Accordingly, we seem to have three relevant conditions – (1) the uncanny affect; (2) imagination; (3) appearance. First, the feeling of uncanniness should be distinguished from more common affective responses such as surprise, fear or mere indifference. Nor is it merely the univocal opposite of what is familiar, in the sense of ‘homely’, ‘cozy’ or ‘intimate’, since not everything which entails a negation of the familiar in this sense will provoke the feeling of the uncanny. As Freud argues, “something has to be added to what is novel and unfamiliar in order to make it uncanny” (1919, p. 221). Secondly, what it is that should be added seems to be contained in the two other conditions: appearance and imagination. Freud illustrates this point with an example taken from his case study of the Rat Man:

In the case history of an obsessional neurotic, I have described how the patient once stayed in a hydropathic establishment and benefited greatly by it. He had the good sense, however, to attribute his improvement not to the therapeutic properties of the water, but to the situation of his room, which immediately adjoined that of a very accommodating nurse. So on his second visit to the establishment he asked for the same room, but was told that it was already occupied by an old gentleman, whereupon he gave vent to his annoyance in the words: ‘I wish he may be struck dead for it’. A fortnight later the old gentleman really did have a stroke. My patient thought this an ‘uncanny’ experience (1919, p. 239).

Here we have all the conditions combined: (1) the uncanny fearful experience, (2) the imagination pertaining to the obsessive wish and (3) reality appearing as if it immediately responded to what was “hitherto regarded as imaginary” (Freud, 1919, p. 242). At this point we can also introduce our previous dialectic between ‘better knowledge’ and ‘belief’. Freud’s first decisive move is to exclude Jentsch’s hypothesis of intellectual uncertainty as an essential factor for the production of the Rat Man’s uncanny experience and seemingly absurd guilt feelings. On such a cognitive account, the Rat Man would be struck by the uncanny precisely because he was, for a brief moment, a spontaneous theoretician of the ‘omnipotence of thoughts’, intellectually unable to rid himself from the illusion that ‘wishes can truly kill’. And the reason for this is precisely because reality appears to correspond with was held to be impossible: better knowledge is suspended because of the qualities of the appearance. Conversely, lifting the veil of appearances would mean to break the spell of the uncanny. However, not only would this idea square rather badly with another of Freud’s (1909, p.229) remarks on the peculiar epistemic position of the Rat Man as being different from “the superstition of uneducated people who feel themselves at one with their belief,” it would also mean that, in the case of Hoffman’s story, our uncanny experience would be due as well to our intellectual uncertainty as regards the true status of the Sand-Man. Yet, as Freud remarks, the story makes it quite clear that the Sand-Man is not really the Sand-man, nevertheless, “this knowledge does not lessen the impression of uncanniness in the least degree” (1919, p. 230). Furthermore, it can even be said that the logic of the uncanny is directly opposed to the logic of intellectual uncertainty. As Dolar (1991) points out, the most uncanny thing is not so much that we are kept uncertain as to the true nature of events, whether or not what happens is really due some unfathomable scenario. On the contrary, what is uncanny is that one knows in advance precisely what is bound to happen, even how and when it will happen … and then, effectively, it really does happen. So here we seem to have attained a first step toward establishing our thesis: the uncanny is an experience that is not altered by a transcendent position of ‘better knowledge’. The Rat Man knows quite well that ‘wishes can’t kill’, that these absurd coincidences mean nothing at all, nonetheless, he is plagued by uncanny feelings and a mythical guilt strangely proportional to a disavowed belief in ‘the omnipotence of thoughts’.

Now the second part of Mannoni’s formula arises: could it be that the uncanny is not only impervious to ‘better knowledge’, but more strongly, that the latter should be seen as its enabling condition? This would mean that in the absence of better knowledge, whenever the distance between myself and the suspended belief disappears, the phenomenon of the uncanny would disappear as well. Again, Freud has noted this point in his subsequent effort, later on in the text, to shift the emphasis from his earlier material definition of the uncanny in terms of the content of the appearance, to a formal definition in terms of the subjective position of those afflicted by the uncanny toward that very same content. Why, for instance, would the prompt fulfillment of Polycrates’ wishes induce an uncanny effect, while the very same appearance of immediate wish-fulfillment in the story of *The Three Wishes* produces none at all? Or whence the uncanniness surrounding the living doll Olympia in Hoffman’s story, while again the same theme of coming-to-life of inanimate objects in most fairy tale stories remains without effect? If it is not the content of the appearance as such that seems to be responsible for the uncanny feeling when reading those stories, then, as Freud argues, we should consider the subjective position from which such stories are read (1919, pp. 245–246). What sets apart, for example, the aesthetic of Hoffman’s story from that of fairy tales is that in the former, at the outset, we are invited to adopt the natural attitude of everyday life. Within this attitude, we know that such figures as the eye-robbing Sand-Man do not exist. In fairy tales, however,

[…] the world of reality is left behind from the very start, and the animistic system of beliefs is frankly adopted. Wish-fulfillments, secret powers, omnipotence of thoughts, animation of inanimate objects, all the elements so common in fairy stories, can exert no uncanny influence here […] (Freud, 1919, p. 250).

Here, precisely, in the enchanted world of fairy tales where the existence of ghosts, resurrection of the dead, miracles and other gruesome plots is assumed, we have nothing to fear. And we have nothing to fear precisely because the difference between better knowledge and belief is temporarily suspended. On the other hand, only if we are invited to the enlightened position of better knowledge, only if we know that wishes are not causally responsible for certain outcomes in the world, do we spontaneously react to those outcomes as if we believed that things could have been different. Similarly, precisely because the Rat Man was “a highly educated and enlightened man of considerable acumen” and therefore “did not believe a word of all this rubbish” (1909, p. 229), did he react with a strange and compulsory form of credulity in the face of the most trivial coincidences. Hence, as Freud aptly notes, we must have “surmounted” certain beliefs in order to be susceptible to the effects of the uncanny (1919, p. 247).

## Conclusion

To conclude our article, we will note three points that are particularly relevant for our discussion. *First*, as was the case in the examples discussed earlier, the logic of the uncanny should be understood in terms of a complex dialectic between better knowledge and belief. Within this dialectic, better knowledge does not form the disjunctive counterpart to belief, but should itself be considered as but one necessary element situated on the same “plane of immanence” (Deleuze, 2005) in conjunction with belief (see also Pfaller, 2014, pp. 41–43). Starting from this general principle, psychoanalytic theory therefore argues for at least two critical points: *first*, pace Dennett and phenomenology, we do not always know what we believe or how things seem to us; *second*, we misrecognize the true function of the alleged distance toward the belief that is expressed through “knowing better” (see Zizek, 1989, pp. 32–33; Pfaller, 2005, p. 118).

*Second*, this idea also enables us to put forward a further, finer distinction between psychoanalytic theory and the aforementioned phenomenological principle of ‘appearance qua appearance’ (cf. supra). According to this principle, beliefs are explained by the properties of the *apparent object*. Because reality appears in such a way as if, for example, it corresponds to the immediate fulfillment of my wishes, I (wrongly) believe that it does. Psychoanalytic theory, however, significantly departs from this picture: if, for instance, the uncanny appears uncanny and I strangely react, despite better knowledge, as if I believed in ‘the omnipotence of thoughts’, then this suspended belief is not primarily due to the properties of the presented object, but first of all to the *act of presentation*. More precisely: on a psychoanalytic account, the uncanny is uncanny not because I confuse appearance with reality, not because I succumb to the illusion and naively follow the appearance, but, on the contrary, precisely because I do *not* follow the appearance, because I am able to recognize the illusion *as* an illusion (or at least believe I can) and therefore picture myself as occupying a transcendent position of better knowledge. In short: exactly when – to put it in Husserlian terms – I do not confuse properties of *what is represented* with the *act of representing*. The illusion, therefore, is not situated at the level of the object, nor is it situated at the level of judgment toward that object, but in how this very act of judgment, and the reflexive self-consciousness of that judgment, contributes to what I ‘objectively believe’.

*Third* and finally, this notion of ‘objective belief’ – i.e., beliefs that are suspended by ‘better knowledge’ – further allows us to confront in a distinctive way what may be considered as the defining gesture of Dennett’s heterophenomenology: the rejection of “the bizarre category of the objectively subjective” (Dennett, 1991, p. 132). According to Dennett, this obscure philosophical notion of the “objectively subjective” should be disposed of because it is once again a clear expression of our obstinate belief in the Cartesian theater. It would correspond to that mythical moment of pure phenomenological ‘givenness’, where the ‘given’ is ‘given’ before being ‘taken’ in one way or another, in a theoretically unencumbered place where consciousness *really* happens. As Dennett notes, such a place might be a comforting image for some philosophers because it preserves the traditional distinction between reality and appearance at the heart of human subjectivity, “the seeming-a-certain-way over and above a believing-that-it-is-that-way” (1991, p. 133). Yet, the examples we discussed point out that this is perhaps not the only way in which “the objectively subjective” can be read. Indeed, instead of believing in “appearances” and not knowing whether or not they correspond to reality, “objective beliefs” display exactly the opposite structure: we know that they are not true, perhaps we even have a thoroughly adequate picture of reality, but the fact that we are nonetheless defined by them, that we act on them, remains undetected (Pfaller, 2014). Hence, from a psychoanalytic view, it is not so much the illusion of the “Cartesian theater”, but the Dennettian theme of “belief in belief” considered as an exhaustive account of subjectivity that should be questioned.

## Author Contributions

JF confirms being a contributor to this work and approved it for publication. SV confirms being a contributor to this work and approved it for publication.

## Conflict of Interest Statement

The authors declare that the research was conducted in the absence of any commercial or financial relationships that could be construed as a potential conflict of interest.

## Footnotes

1 For a representative sample of the different discussions running through this debate, see the collection of essays in Dreyfus and Kelly (2007).

2 This is indeed the proposal we can read in the works of Sartre and – even earlier – Politzer: to release psychoanalysis from its “mechanistic cramp” (Sartre, 1979, p. 32) or “abstract” and “realist” exigencies (Politzer, 1994) – for an excellent overview of Politzer’s somewhat forgotten critique, see Bianco (2016).

3 For a defense of this reading, see, e.g., Moore (1988); Kitcher (1992) and Livingstone-Smith (1999).
